# Soil organic nitrogen: an overlooked but potentially significant contribution to crop nutrition

**DOI:** 10.1007/s11104-021-04860-w

**Published:** 2021-02-18

**Authors:** Soudeh Farzadfar, J. Diane Knight, Kate A. Congreves

**Affiliations:** 1 Department of Plant Sciences, University of Saskatchewan, Saskatoon, SK S7N 5A8 Canada; 2 Department of Soil Science, University of Saskatchewan, Saskatoon, SK S7N 5A8 Canada

**Keywords:** Plant nutrition, Organic nitrogen, Uptake and assimilation, Agriculture

## Abstract

**Background:**

For more than a century, crop N nutrition research has primarily focused on inorganic N (IN) dynamics, building the traditional model that agricultural plants predominantly take up N in the form of NO_3_^−^ and NH_4_^+^. However, results reported in the ecological and agricultural literature suggest that the traditional model of plant N nutrition is oversimplified.

**Scope:**

We examine the role of organic N (ON) in plant N nutrition, first by reviewing the historical discoveries by ecologists of plant ON uptake, then by discussing the advancements of key analytical techniques that have furthered the cause (stable isotope and microdialysis techniques). The current state of knowledge on soil ON dynamics is analyzed concurrently with recent developments that show ON uptake and assimilation by agricultural plant species. Lastly, we consider the relationship between ON uptake and nitrogen use efficiency (NUE) in an agricultural context.

**Conclusions:**

We propose several mechanisms by which ON uptake and assimilation may increase crop NUE, such as by reducing N assimilation costs, promoting root biomass growth, shaping N cycling microbial communities, recapturing exuded N compounds, and aligning the root uptake capacity to the soil N supply in highly fertilized systems. These hypothetical mechanisms should direct future research on the topic. Although the quantitative role remains unknown, ON compounds should be considered as significant contributors to plant N nutrition.

## Introduction

The anthropogenic nitrogen (N) footprint is estimated at up to 41 kg N capita^− 1^ yr^− 1^, of which food production represents the single largest contribution to the footprint (Leach et al. 2012). Approximately 100–125 Tg yr^− 1^ of synthetic inorganic N (IN) is applied to agricultural and horticultural systems globally (Fowler et al. 2013). Due to the dependence of modern agriculture on the production and use of IN fertilizers, food production has dramatically altered the global N budget (Fowler et al. 2013). As much as 50 % of N applied is not used by the crop (Cameron et al. 2013) and, after a series of N transformations, is at risk of movement to aqueous or atmospheric environments where it can be a serious pollutant. This in turn alters regional biogeochemical N cycles as well as the cycling of carbon (C) and other elements (Galloway et al. 2003). Agriculture’s reliance on IN fertilizers stems from the dogma that plants take up N predominantly as NO_3_^−^ and NH_4_^+^. Advancing the understanding of N contributions in forms other than IN to plant N budgets will provide a necessary pathway for reducing the overreliance of agriculture on IN fertilizers, help develop agricultural systems that use N more efficiently and reduce agriculture’s negative impact on the environment (Wang et al. 2018).

Improving crop N use efficiency (NUE) is one means of reducing N losses from a system. Generally, agronomists advocate for the implementation of “4R” practices to improve NUE, which involves applying fertilizer at the right rate, right time, right source and right placement in the soil (Sposari and Flis 2017). In practice however, improving crop NUE remains a major challenge (Masclaux-Daubresse et al. 2010; Qiao et al. 2015) and research is needed to provide a deeper understanding of soil-plant N cycling to enhance crop NUE. While there has been a shift in the ecological plant N nutrition model over the last several decades from a focus on IN uptake towards a model that also includes various forms of ON compounds, it has only recently taken root in agricultural systems (Paungfoo-Lonhienne et al. 2012). Due to the long held the belief that NO_3_^−^ and NH_4_^+^ are the only major N sources for crops (Haynes 1986; von Wirén et al. 1997; Jackson et al. 2008), most agricultural studies measure soil IN dynamics rather than soil ON compounds (Franzluebbers et al. 1995; Korsaeth et al. 2002; Geng et al. 2015–to name just a few). Many agricultural NUE indices entirely ignore soil ON contributions i.e., calculations for agronomic efficiency, recovery efficiency, uptake efficiency, apparent recovery, etc. (Dobermann 2007); various soil N cycling simulation models used for agricultural systems do not contain subcomponents for simulating soil ON uptake by plants (Addiscott and Whitmore 1987; Parton et al. 1998; Liu et al. 2011); agricultural fertilizer recommendations are based on soil IN levels and do not consider soil ON compounds. The significance of ON to agricultural crop production remains little understood but could contribute to crop NUE dynamics.

**Fig. 1:**
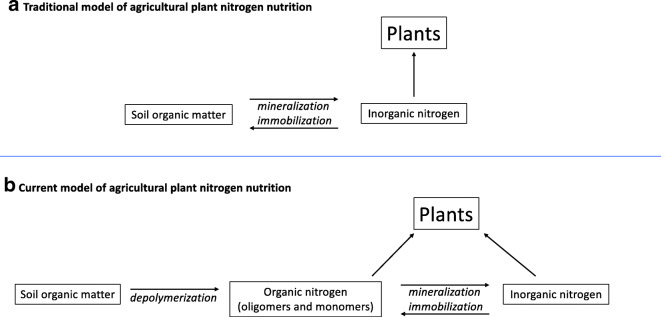
Traditional and emerging model of plant N nutrition in agricultural contexts. The figure is inspired by Schimel and Bennett (2004) and Schmidt et al. (2014). The italicised words represent microbial processes

Mainstream agricultural systems are starting to incorporate practices that resemble organic production, such as integrating cover crops and green manure, managing crop residues and incorporating organic amendments to improve soil health and optimize crop nutrition (Norris and Congreves 2018). These practices capitalize on the decomposition of organic materials and the cycling of nutrients to reduce reliance on synthetic N inputs. Soil ON compounds include proteins, peptides, ureides, and amino acids (AAs), some of which are dissolved in soil water and free, while others are complexed to soil minerals. Other N-containing compounds include nucleobases, nucleosides or nucleotides, and secondary metabolites, among others. The importance of the soil ON reservoir in supplying IN that subsequently is used by plants has long been recognized by ecologists, but the mechanisms explaining how and why agricultural crops acquire and utilize ON is less well studied than plants in natural ecosystems. Today, we understand that plants can take up a variety of N forms, be that inorganic or organic (as organic oligomers or monomers) (Schmidt et al. 2014). The recognition that ON is taken up by plants is not new, but its significance for agricultural crops such as wheat, barley, maize, clover and sugarcane, has only recently been reported (Jämtgård et al. 2008; Reeve et al. 2009; Brackin et al. 2015; Czaban et al. 2016; Hill and Jones 2019; Czaban and Rasmussen 2019; Enggrob et al. 2019; Rasmussen et al. 2019). Agricultural systems typically receive external N inputs and tend to be more nutrient rich than natural systems—imposing very different conditions on N cycling processes than in natural systems. As such, there is arguably a crucial research gap centered on the relative importance of the availability, uptake mechanisms and plant use of ON sources in *agricultural systems*. This calls for a deeper understanding of ON uptake in agricultural crops—especially considering the urgent need to improve crop NUE. In this article, we discuss recent advances in understanding crop ON uptake that have contributed to the current understanding of agricultural plant N nutrition (Fig. 1)—beginning with a summary of the historical progress.

## Advancements in understanding the contribution of organic nitrogen to crop nutrition: a historical sketch

The idea that plants have the capacity to take up ON compounds, such as AAs, goes back to the 19th century, and was comprehensively reviewed by McKee (1962). By the beginning of the 20th century studies were emerging with reports that plants were able to use different ON forms for growth (Hutchinson and Miller 1912; Schreiner and Skinner 1915). Most of the early work focused on direct uptake of AAs. In the 1950s and 1960s there were several reports of plants incorporating exogenously applied AAs into proteins in tissues, from which it could have been concluded that plants can take up AAs (e.g., Boroughs and Bonner 1953; Webster 1954; Reinhold and Powell 1958). During this period, it was also shown that mycorrhizal fungi can take up AAs and transfer N from these compounds to their host plants (Melin and Nilsson 1953). Between the end of the 1950s and the early 1970s, several studies characterized AA uptake by a variety of plant tissues such as carrot slices (Birt and Hird 1956) and leaf fragments (Parthier et al. 1964), excised plant organs such as roots (Wright 1962), root tips (Newton 1974) and scutella (Stewart 1971), and cultured plant cells (Hart and Filner 1969).

Importantly, the uptake of AAs by intact plants was convincingly shown by Bollard (1966). Using aseptic cultures of *Spirodela olighorhiza*, it was clearly demonstrated that asparagine, glutamine, glutamic acid and the metabolically related γ-aminobutyric acid could all serve as the only N source, producing copious growth of the duckweed (Bollard 1966). In this study, it was also shown that some dipeptides could also serve as N source for plants (Bollard 1966). In the 1970s, the characteristics of AA transport processes in plants were further elucidated. For example, in a series of studies using axenic cultures of another duckweed, *Spirodela polyrhiza*, the stereospecific uptake, accumulation, and metabolization of exogenously supplied AAs, and competition between AAs for uptake have been demonstrated (Borstlap 1972, 1974, 1975, 1977). A dichotomy in transport specificity with neutral and acidic AAs on one hand, and basic AAs on the other was also uncovered early (Borstlap 1974), and largely confirmed later (Kinraide 1981; Borstlap et al. 1985, 1986; Schobert and Komor 1987; Heremans et al. 1997). Interestingly, after the discovery and characterization of many AA transporters in plants, this dichotomy is by and large still valid (Tegeder and Rentsch 2010; Tegeder 2012).

The first gene of a plant AA transporter was cloned in 1993 in Jörg Riesmeier’s laboratory (Frommer et al. 1993) and independently in the lab of Daniel Bush (Hsu et al. 1993). Since then, dozens of genes of (putative) AA transporters have been identified in each of several plant species, from more than 60 in *Arabidopsis* and 72 in potato, up to 189 in soybean, and even as much as 283 in wheat (Cheng et al. 2016; Ma et al. 2016a; Wan et al. 2017; Yao et al. 2020). It is well known that plants possess high affinity as well as low affinity AA transporters. Typically, the high affinity (= low K_m_) transport systems have much lower V_max_ than the low affinity (= high K_m_) systems (Borstlap 1977; Soldal and Nissen 1978; Schobert and Komor 1987; Fischer et al. 2002; Jämtgård et al. 2008; Svennerstam et al. 2011). The occurrence of low- and high affinity transporters in cells is quite general (Levy et al. 2011; Eide 2012), but why plants have AA transporters of widely different affinities is still an enigma.

A number of important contributions on how plants use ON sources in the soil were developed in the 1990s (Kielland 1994; Näsholm et al. 1998, 2000; Kielland et al. 2007; Jämtgård et al. 2008). Several studies confirmed that AAs are absorbed not only by ectomycorrhizal (EcM), ericoid mycorrhizal (ErM) and arbuscular mycorrhizal (AM) plants, but also by non-mycorrhizal plants (Chapin et al. III 1993; Näsholm et al. 1998, 2000; Persson and Näsholm 2002; Leinweber et al. 2013) were the first to report that ON was the preferred source of N for growth of a non-mycorrhizal arctic sedge (*Eriophorum vaginatum*), resulting in more N and biomass accumulation than when the plant was supplied with IN and that the reverse was exhibited by barley (*Hordeum vulgare*). Based on this early work, ON was originally considered a relatively minor source of N for plants, and ON uptake was assumed to only occur in natural environments with low IN availability, i.e., arctic or forested ecosystems rather than agricultural systems (Näsholm et al. 1998). This assumption was born out of the differences in soil IN levels (agricultural soils tend to have higher soil IN levels than natural ecosystems) and may explain why ecologists have long accepted that ON forms are important N sources for plants, while agronomists have long held the belief that NO_3_^−^ and NH_4_^+^ are the only N sources for crops. Nevertheless in agricultural systems ON forms constitute 80 to 90 % of the total soil N pool, except immediately after IN fertilizer application when the fraction of total soil N as IN increases (Schulten and Schnitzer 1998; Liu et al. 2018). Further, the pool of small ON compounds in the soil solution can be as large as those of IN (Warren 2014). Although the proportion of total soil N as ON might be lower in agricultural systems than in natural ecosystems(Schimel and Bennett 2004), seminal reviews by Näsholm et al. (2009) and Paungfoo-Lonhienne et al. (2012) have inspired the question: how much does soil ON contribute to plant nutrition in agricultural systems?

In parallel to the developments in understanding how plants acquire and use ON, advancements in soil science techniques expanded capabilities to study ON dynamics at the soil-plant interface, namely stable isotope techniques and microdialysis (amongst others such as nuclear magnetic resonance, spectroscopic analyses etc. which are beyond the scope of this review). Stable isotopes (^13^ C, ^15^ N, ^18^O, ^2^H) are naturally occurring forms of elements that can be used as tracers to understand how elements are utilized, stored, transformed, and lost. Without isotopes, understanding the fates of these elements in agricultural systems is difficult, due to the many different processes that affect a compound’s movement and transformation within and across plant-soil-atmosphere boundaries. In bulk stable isotope analysis (BSIA), the bulk plant material is assayed for the stable isotope—which enables tracing the element of interest as it moves from one component to another, e.g., from a labelled compound in the soil into bulk plant tissues.

To distinguish between intact AA uptake and N taken up after AAs are mineralized, labeled ^13^ C and ^15^ N compounds have been traced from the soil to plant tissues using BSIA (Rasmussen and Kuzyakov 2009; Rasmussen et al. 2010). However, plant ^13^ C lost through respiration and transpiration can be recaptured by photosynthesis or root uptake of HCO_3_^−^, which challenges the interpretation of plant tissue bulk ^13^ C results (Rasmussen and Kuzyakov 2009). Moreover, the dual-labeling and BSIA approach can be confounded by uptake of labeled inorganic C or N produced by mineralization of labeled AAs (which can happen quickly in the soil), producing inconclusive results (Rasmussen et al. 2010). As such, BSIA alone cannot differentiate between uptake of intact AAs and uptake of N molecules that were transformed prior to uptake, and may lead to either overestimation (Sauheitl et al. 2009; Rasmussen et al. 2010), or underestimation of uptake due to respiratory loss of ^13^ C (Czaban et al. 2016; Dion et al. 2018).

To reduce bias that may arise when using BSIA to understand plant ON uptake, compound-specific isotope analysis (CSIA) has emerged as a more powerful analytical tool (Charteris et al. 2016; Ohkouchi et al. 2017). In CSIA, it is the labelled compound itself that is analyzed in the plant material, and this enables tracing a particular labelled compound from the soil into the plant. This approach considerably reduces the overestimation of intact AA uptake, relative to BSIA (Xu et al. 2006, 2008). Although CSIA is useful, it is not without problems. Low molecular weight ON compounds are rapidly metabolized in the soil, and also in the root post-uptake, which has profound implications for interpreting the results in isotope labelling studies (Wanek et al. 2010; Hu et al. 2017, 2020; Noll et al. 2019; Warren 2019). Many AAs in soil solution have residence times in the order of 10–30 minutes (Warren 2019; Hu et al. 2020), which constrains the duration of experiments to ideally < 10–30 minutes. Hence, matching the timing of incubations to the rapidity of soil N cycling can provide more accurate estimates of N recovery from ON forms for sample analysis with both BSIA and CSIA (Wanek et al. 2010; Hill and Jones 2019; Warren 2019).

Using dual-labelling (^13^ C and ^15^ N) to trace large ON compounds brings another set of challenges (Enggrob et al. 2019). Depolymerization of large ON compounds often occurs before the ON source is readily available to plants. As such, a short experimental period of tracer pulsing may not allow sufficient time to capture depolymerization *and* subsequent plant uptake of the depolymerized peptides or AAs.

Drawbacks of conventional soil sampling methods, such as destructive bulk soil sampling, sample clean-up, sample degradation or transformation through extended handling, along with traditional extraction methods are potential sources of misinterpretation in soil N studies (Buckley et al. 2020). For instance, soil sieving and conventionally used extraction methods (water extraction, and KCl extraction) may modify N pools within a soil sample by damaging structures such as fine roots and hyphae, mineralizing ON by which IN will be overestimated (Inselsbacher et al. 2011; Buckley et al. 2020). Microdialysis is used to overcome these drawbacks; it is a membrane-based sampling technique that takes advantage of on the concentration gradient across a semi-permeable barrier. The technique is well established and extensively used in biomedical research, but introduced as a method to study soil N dynamics more recently (Inselsbacher et al. 2011; Inselsbacher and Näsholm 2012). It can be used to measure soil N pools at high spatial and temporal resolution, isolate soil N compounds, and in monitoring changes of N concentrations at the root-soil interface. In practice, small probes (0.5 mm diameter) are fitted with permeable membranes at root-relevant scales, resulting in minimal soil disturbance (Inselsbacher et al. 2011; Buckley et al. 2020). Importantly, due to their small size and performance as a sink for free-moving compounds, the microdialysis probes might, to some extent, mimic plant N uptake via diffusion (Inselsbacher and Näsholm 2012). In contrast to the results of conventional extraction methods, microdialysis has shown that AAs might dominate soil N supply even in agricultural soils (Inselsbacher and Näsholm 2012; Oyewole et al. 2014; Brackin et al. 2015; Buckley et al. 2020). Even though di- and tripeptides turn over rapidly in soil, samples have been successfully collected using microdialysis (Hill et al. 2012; Jones and Kielland 2012; Jämtgård et al. 2018).

## Soil nitrogen transformations and availability

Soil organic matter (SOM) dynamics are intricately related to soil N transformations, as C and N are both components of SOM and subjected to transformation via microorganism activity (Cotrufo et al. 2013; Lehmann and Kleber 2015; Kallenbach et al. 2016). Despite widespread agreement that SOM serves as a crucial storehouse and supplier of N for microorganisms and plants, soil ON dynamics remain poorly understood and not fully characterized (Warren 2014).

Based on the current understanding of SOM, soil organic compounds are conceived as a continuum from in-tact organic material, to large biopolymers, small biopolymers, and monomers (Lehmann and Kleber 2015). Most of soil N (> 80 %) is in polymeric form and generally believed to be inaccessible for direct uptake by microbes or plant roots (Schulten and Schnitzer 1998; Geisseler et al. 2010; Liu et al. 2018). Complex organic compounds must first undergo a cascade of enzymatic reactions, and the resulting products can be mineralized to IN forms. The IN forms may then be assimilated (“immobilized’’) by microbes—a process known as the mineralization-immobilization-turnover (MIT) route. Recent research has clearly demonstrated, however, that small ON products (AAs and other ON compounds) can also be assimilated directly by soil microbes (Geisseler et al. 2010).

The basic understanding of soil N mineralization/immobilization has not changed for several decades (Stanford and Smith 1972; Robertson and Groffman 2007): organic materials with C:N ratios of approximately 25:1 or less result in net soil N mineralization, whereas those with higher C:N ratios result in net soil mineralization. This phenomenon is explained by the relative differences in the microbial requirements of N and C to fuel decomposition. During soil N mineralization, ON in soil organic matter is ammonified by soil microbes into ammonia (NH_3_) and ammonium (NH_4_^+^)—a process which is often quickly followed by nitrification in agricultural soils, converting soil NH_4_^+^ to other soil IN forms of NO_2_^−^ and NO_3_^−^.Soil N may be lost in denitrification processes by which NO_3_^−^ is converted into gaseous forms of NO_2_, N_2_O, and N_2_. Nitrogen loss may also occur via NH_3_ volatilization, by the leaching of mobile nitrogenous compounds such as NO_3_^−^ or dissolved ON (DON), and by surface runoff and erosion.

Due to its size and negative charge, NO_3_^−^ is readily mobile in soil and easily leaches downward into groundwater—a risk not shared by NH_4_^+^. Dissolved ON presents a similar risk as NO_3_^−^ to leaching and can account for significant N loss (Neff et al. 2003). In a review of 16 studies that examined leaching losses, DON losses accounted for 26 % of the total soluble N losses (NO_3_^−^ plus DON) from agricultural ecosystems (van Kessel et al. 2009). Nonetheless, soil ON compounds, ranging from simple labile forms like AAs to complex and insoluble aromatic polymers, are generally less mobile in the soil solution than NO_3_^−^ (Miller and Cramer 2005; Jämtgård et al. 2008).

In agricultural soils, manure, crop residues, roots and root exudates are the main sources of ON, along with dead organisms such as bacteria, archaea, fungi, invertebrates, and animal tissues. The proteins in these sources of ON are hydrolyzed into AAs, and together with their oligomers and polymers, they are the most prevalent forms of ON in agricultural soils. Amino acids comprise approximately 20–30 % of the total N in soil organic matter (Stevenson 1994)—which is higher than soil IN levels that typically range from 10–20 % of total N. Up to 99.5 % of AAs in soil are contained in polymeric N-containing compounds such as protein–humic complexes and peptides while free AAs constitute only 0.5 % of the total AAs in surface soils (Roberts and Jones 2008). The AAs bound in peptides and proteins are the most abundant fraction of AAs and considered as the key source for releasing free AAs (Jämtgård et al. 2008, 2010; Näsholm et al. 2009).

Dissolved ON compounds are typically categorized into three classes according to molecular weight (Farrell et al. 2011). The LMW class includes AA and oligopeptides composed of up to ca. 8–12 AAs with a molecular weight less than 1 kDa; the intermediate class (medium molecular weight, MMW) consists of peptides and proteins with molecular weights between 1 and 100 kDa; the high molecular weight (HMW) class is comprised of oligomers and polymers of AAs with a molecular weight higher than 100 kDa. The functional and ecological differences in these classes, directly or indirectly, are related to their molecular weight (Warren 2014). For instance, LMW DON turns over rapidly (1 to 12 hours), while MMW and HMW DON turns over more slowly (days to months) (Jones et al. 2004; Warren 2014). The mineralization of a soil protein solution containing a mixture of 65, 75 and 120 kDa HMW compounds was ca.. 20 times slower than the mineralization rate of free AAs (Jan et al. 2009; Farrell et al. 2011) showed that the HMW fractions were the most abundant form of DON in grasslands soils, followed by the LMW fractions, while the MMW fractions were the least abundant. The depolymerization of HMW to LMW ON fractions is considered a bottleneck in soil N cycling and thereby is a major control of the availability of N to plants and microbes (Schimel and Bennett 2004; Jan et al. 2009). Abundance of DON follows a seasonal pattern in temperate environments presumably in response to increasing soil temperatures. In spring, an increase in total DON occurs when soil microbes become more active (Farrell et al. 2011; Hill et al. 2011a). This coincides with a peak in soil protease activity releasing free AAs which can be taken up by microbes and plants (Raab et al. 1999; Bardgett et al. 2005; Farrell et al. 2011). The LMW and HMW fractions are believed to be the functional pools of DON; the HMW fractions account for seasonal changes in DON abundance, whereas the relative abundance of LMW peptides only modestly change with seasons, and are considered as a pool of readily-assimilatable N for soil microbes and plants (Farrell et al. 2011).

Among the LMW compounds, quaternary ammonium compounds (QACs) are abundant, representing 1–28 % of the nonpeptide small ON pool (Warren 2013). These compounds include betaine, proline betaine, ectoine, hydroxyectoine, and hercynine have key roles in osmotic adjustment and stress tolerance of soil microbes (Warren 2013, 2014). Both the nonmycorrhizal *Banksia oligifolia* and mycorrhizal wheat (*Triticum aestivum*) took up intact molecules of QACs from hydroponic culture, and wheat from soil into which QACs were injected (Warren 2013). D-amino acids are key components of peptidoglycan in bacterial cell walls and can be introduced to soil through bacteria, faeces, eukaryotic biomass, antibiotics, synthetic pesticides and through racemization of L-amino acid forms (Vranova et al. 2012). They appear to undergo slower mineralization than L-forms (Hill et al. 2011b; Vranova et al. 2012) and can be taken up by soil microbes and plants directly when introduced to the system, although the size and significance of natural concentrations of D-amino acids in soils has been complicated by experimental limitations (Vranova et al. 2012).

Microbial necromass-N accounts for > 60 % of soil ON and is mainly composed of poly-amino compounds, of which proteins are the most abundant class (Schulten and Schnitzer 1998; Roberts and Jones 2012; Hu et al. 2020). Polymers of amino sugars make up the outer layer of bacterial and archaeal cell walls and contain large amounts of glucosamine (Roberts and Jones 2012; Hu et al. 2020). Galactosamine is an isomer of glucosamine that is embedded in extracellular polymeric substances such as glycol-conjugated proteins or lipopolysaccharides attached to microbial cell walls in soil and accounts for 17 to 42 % of total soil amino sugars (Joergensen 2018; Hu et al. 2020). Glycoproteins such as bacterial peptidoglycan and fungal chitin must undergo some decomposition prior to being taken up by soil microbes (Hu et al. 2020). For example, MMW cell wall muropeptides produced during the decomposition of bacterial peptidoglycan can be directly utilized by microbes without being further depolymerized to LMW muramic acid and other monomeric amino compounds (Hu et al. 2020). However, soil proteins and glycoprotein must depolymerize to LMW monomeric amino compounds like free AAs and amino sugars to be utilized by microbes (Hu et al. 2020). Their products, at various degrees of decomposition (from MMW muropeptides to amino sugars, and meso-diaminopimelic acid, and D-amino acids) contribute to the soil ON pool together with proteinogenic L-amino acids (Warren 2014; Hu et al. 2020). The microbial cell walls are as recalcitrant as proteins, but their turnover largely depends on soil physicochemical and soil biological conditions, which can be tentatively ascribed to their glyco-conjugates (Warren 2014).

## Organic nitrogen uptake and assimilation by plants

Nitrogen compounds, either IN or ON, must interface directly with the surface of roots to be taken up by plants. Movement to roots occurs via mass flow, diffusion, and root interception—the first two being the more dominant pathways. Plant transpiration is the main driver of plant N acquisition (Oyewole et al. 2014) by moving water soluble N forms towards plant root surfaces, and by controlling a concentration gradient of N-containing compounds from root surfaces out into the soil. Both IN and ON are taken up in a concentration dependent manner under the control of transporters, and uptake is mediated by high and low affinity transport systems. However, differences in molecular size and charge between soil IN and ON compounds result in different diffusion coefficients and hence the supply of AAs to plant roots via diffusion tends to be considerably lower than NO_3_^−^. In the soil, diffusion coefficients for NH_4_^+^, lysine, glycine and glutamate are lower than that of NO_3_^−^ by factors of 122, 292, 36, and 26, respectively; however, glycine and glutamate have diffusion rates 3.4- and 4.7-times higher than NH_4_^+^ (Owen and Jones 2001; Miller and Cramer 2005).

Plant root transporters for the uptake of IN and ON are well described elsewhere (Rentsch et al. 2007; Tegeder and Masclaux-Daubresse 2018), and readers are referred to these sources for further information. The uptake of strong ions NO_3_^−^ and NH_4_^+^ result in alkalinization and acidification of the root environment, respectively; however, this is not the case for AA uptake. Although AA are taken up and accumulated by plants through a proton symport mechanism (Bush 1993; Boorer et al. 1996; Borstlap and Schuurmans 2000), this will not result in a sustained alkalinization of the root environment. Only a slight, short-lived alkalinization near the root surface may occur, which is rapidly neutralized by the activity of the plasma membrane proton pump (H^+^/ATPase) (Kinraide and Etherton 1980).

In recent years, a number of stable isotope studies have demonstrated ON uptake by crop species—and Table 1 is presented as a resource to direct readers to this body of literature. Readers may find this list useful for informing methodology, selecting crop species to study, and in identifying current gaps. For example, most of these studies have focused on uptake of individual AAs, whereas gaps clearly remain for a range of other, larger ON compounds. Also, many studies have focused on wheat, whereas a smaller proportion have evaluated horticultural crops (Table 1). It is not surprising that the results are overall variable (Table 1), as uptake is a function of the specific ON source, the concentration supplied, the duration and specific conditions of the experiment, and the crop species studied. The wide range in results emphasizes the need to carefully select the concentration of the ON compound supplied to the plant, and the study duration. For example, in the soil solution, individual AAs typically have concentrations ranging from high nanomolar to low micromolar levels, but a number of these studies exposed plant roots to much higher concentrations of ON, in some cases approaching millimolar levels (Table 1). The high uptake rates associated with high initial ON concentrations may or may not be replicated under field conditions. As indicated by the lack of field-based studies in Table 1, moving beyond lab-based or controlled-environment studies to field studies presents considerable challenges. However, it will be nonetheless necessary to quantitatively characterize the role of ON in agricultural crops.

**Table 1:** Recent research demonstrates the uptake of organic nitrogen compounds by various crop species

Organic N compound	Crop	Technique used*	Concentration in root media (mg N L^− 1^)	Incubation time with labeled ON in different media	ON uptake rate (µmol g^− 1^ DM h^− 1^)	Reference
Alanine	*Cucumis sativus*	CSIA, N based	0.64 to 19.10	2 h in nutrient solution	0.3 to 3.5	Dion et al. (2018)
		CSIA, C based	0.64 to 19.10		0.1 to 1.5	
		BSIA, N based	0.64 to 19.10		14.5 to 17.0	
		BSIA, C based	0.64 to 19.10		2.0 to 10.5	
Glycine	*Triticum aestivum*	CSIA, N based	14 to 28	60 h in nutrient solution	5.1 to 11.5	Gioseffi et al. (2012)
		CSIA, C based	14 to 28		1.7 to 3.5	
Glutamine	*Triticum aestivum*	CSIA, N based	14 to 28		5.0 to 8.40	
		CSIA, C based	14 to 28		1.5 to 2.1	
Glycine	*Oryza sativa*	BSIA, N based	5 to 1200	21 days in sterilized soil		Cao et al. (2013)
Glycine	*Triticum aestivum*	BSIA, C based	0.14 to 14	24 h in agricultural soil		Reeve et al. (2009)
Asparagine	*Trifolium repens*	BSIA, N based	0.11 to 17.50	6 weeks in nutrient solution	0.20 to 32.5	Czaban et al. (2016)
		CSIA, N based	0.11 to 17.50		0.04 to 4.8	
Glycine	*Brassica campestris*	BSIA, N based	0.35 to 21	4 h in nutrient solution 12 h in nutrient solution	0.04 to 15.5	Cao et al. (2015); Ma et al. (2016b)
L-alanine	*Triticum aestivum*	CSIA, N based	0.14	5 h in MS agar medium	0.60 to 1.20	Hill et al. (2011b)
D-alanine	*Triticum aestivum*	CSIA, N based	0.14		0.24 to 0.36	
L-trialanine	*Triticum aestivum*	CSIA, N based	0.14		0.26 to 0.34	
D-trialanine	*Triticum aestivum*	CSIA, N based	0.14		0.01 to 0.07	
Glycine	*Saccharum officinarum*	CSIA, N based	0.42 to 42	4 h in agricultural soil	26.2 to 29.9	Brackin et al. (2015)
Glycine	*Ocimum basilicum*	BSIA, N based	0.14 to 24.50	2 weeks in a mixture of sand and soil	0.10 to 1.00	Warren (2009)
Alanine	*Zea mays*	CSIA, C based	0.70	6 h in soil filled rhizotubes		Moran-Zuloaga et al. (2015)
Alanine	*Lupinus albus*	CSIA, C based	0.70			
Protein ON	*Zea mays*	CSIA, C based	160	48 h in agricultural soil		Enggrob et al. (2019)
^*^ Where applicable, two isotope techniques are included: CSIA, Compound-specific isotope analysis; BSIA, Bulk stable isotope analysis, because the two techniques can produce different ON uptake results and interpretations.						

Stable isotope labeling studies as a whole have contributed valuable information on various aspects of ON uptake and assimilation. Dion et al. (2018), demonstrated that cucumber (*Cucumis sativus*) exposed to lower soil IN:ON ratios acquired more of their N from AA sources compared to plants exposed to higher IN:ON ratios. Similar results were reported for white clover (*Trifolium repens*) (Czaban and Rasmussen 2019) and wheat (Gioseffi et al. 2012). However, the preferential uptake of IN when it is in abundant supply does not mean that ON is completely excluded; indeed, plant uptake of ON still occurred in the presence of soil IN (Czaban et al. 2016). Rather, it seems that plants may rely more heavily on ON sources under low-input agricultural systems with low inherent soil IN levels, than under highly fertilized systems. In production systems that receive large amounts of IN-fertilizer, there can be a potential mismatch between the supply of N and the capacity of the roots to take up N. For example, in a fertilized sugarcane field in Queensland studied by (Brackin et al. 2015), fluxes of ON more closely matched root uptake capacity than that of IN.

Uptake of N derived from complex organic compounds, such as proteins, can exceed uptake of N derived from AAs. For example, maize (*Zea mays*) supplied with triple labelled complex organic compounds (> 100 kDa ON) took up similar or higher amounts of N than when supplied with single AAs (Enggrob et al. 2019). In their study, it was estimated that 20–30 % of the N uptake occurred in the organic form, and it was inferred that the importance of plant ON uptake increases when N is derived from complex molecules compared to single AAs. In comparing the mineralization of labelled > 100 kDA ON with vs. without maize, more AAs which were derived from of the labelled-ON source remained in the soil with vs. without maize. An explanation for this result may be that rhizosphere microorganisms increase anabolic utilization of ON, relative to those in the bulk soil (Enggrob et al. 2019).

Plants also possess specific urea transporters that either actively or passively transport urea into the plant (Witte 2011), efficiently hydrolyze urea, and can use urea as sole N source (Nacry et al. 2013). Urea is an intermediate of plant arginine catabolism involved in N remobilization from source tissues (Witte 2011). In addition, plants are able to uptake and utilize peptides, QACs and D-enantiomers of AAs (Bollard 1966; Breitkreuz et al. 1999; Komarova et al. 2008; Hill et al. 2011b; Soper et al. 2011; Warren 2013, 2014). Di- and tripeptides are the intermediaries between polymeric and monomeric DON in the soil N cycle. Although levels of individual dipeptides in soil are an order of magnitude lower than those of free AAs, they degrade at speeds similar to free AAs. Free AAs and dipeptides are transported into roots (Komarova et al. 2008), as indicated by the presence of intact dipeptides in plants grown in axenic culture (Soper et al. 2011) and the uptake of isotopically-labelled peptides in the field (Hill et al. 2011a). Overexpression of di- and tripeptide transporters in *Arabidopsis* mutants resulted in greater biomass and N content than wild-type plants when supplied with peptides as a sole N source (Komarova et al. 2008). Such results not only provide evidence of intact peptide uptake but outline the role of peptide transport and utilization for plant growth. D-AAs, as essential components of bacterial peptidoglycan, are known to account for a significant but highly variable proportion of AAs in hydrolyzed soil (Wichern et al. 2004; Amelung et al. 2006). Because certain D-AAs like D-serine have phytotoxic effects at high concentrations, it was assumed that they would not represent an important N source for plants (Erikson et al. 2004, 2005). However, Hill et al. (2011b) reported that wheat plants took up and assimilated D-alanine when supplied at realistic soil solution concentrations; and they did so in preference to NO_3_^−^. Overall, several lines of evidence indicate that the soil ON pool accessible by plants is highly diverse, and that ON forms other than AAs can serve as potential N sources. It is prudent that plant uptake research efforts not focus solely on free AAs to the exclusion of other ON source; similarly advised by Warren (2014) when reviewing soil organic N dynamics. Warren (2014) sagely points out that the understanding of ON nutrition to plants cannot move forward until the design and interpretation of experiments is informed by more complete knowledge of ON molecules in soil solution.

**Fig. 2:**
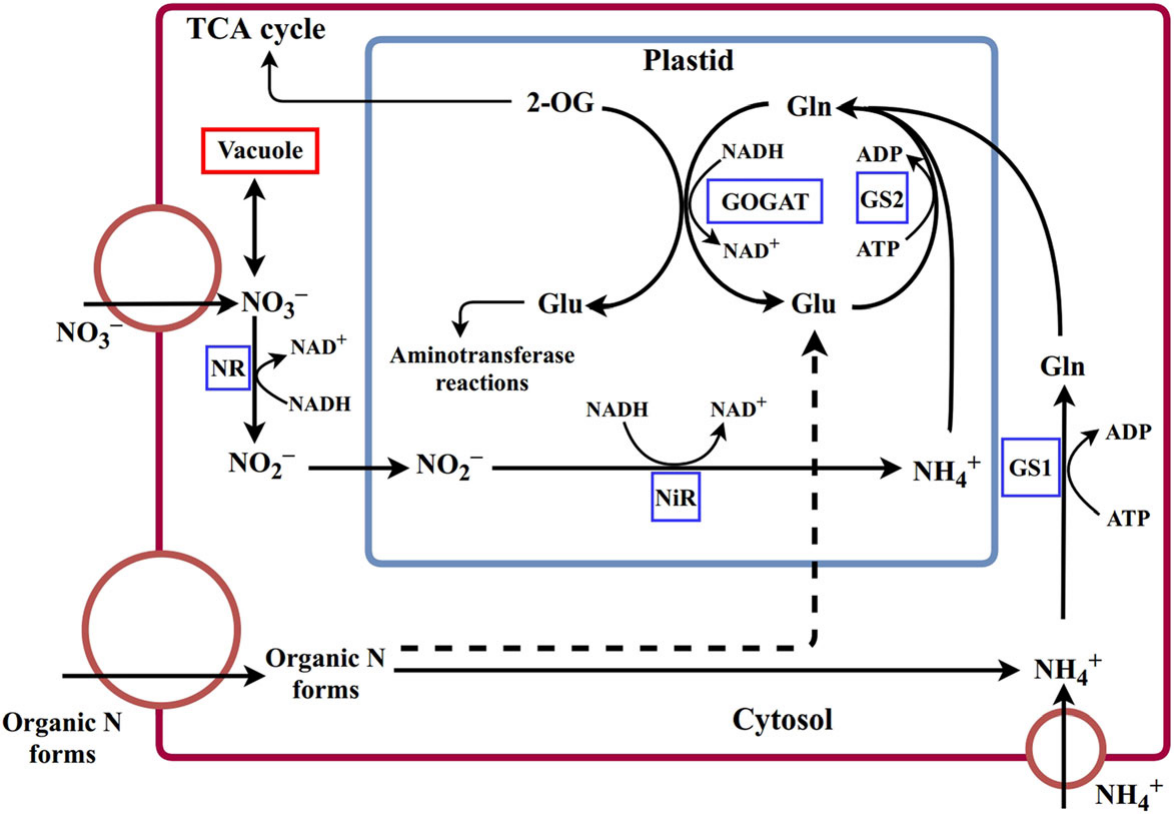
Nitrogen uptake and assimilation. Schematic overview of nitrate uptake and assimilation into amino acids occurring in the cytosol and the plastid of a root cell. Abbreviations: NR, nitrate reductase; NiR, nitrite reductase; GS_1_, cytosolic glutamine synthetase; GS_2_, plastidial glutamine synthetase; Gln, glutamine; NAD(H)-GOGAT, plastidial glutamate oxoglutarate aminotransferase; GDH, glutamate dehydrogenase; Glu, glutamate; 2-OG, 2-oxoglutarate and TCA cycle, tricarboxylic acid cycle. Circles represent transporters. The dashed line indicates several stages involved in the conversion of organic N forms to Glu

Once inside plant root cells, ON compounds are catabolized in the cytosol releasing NH_4_^+^ and intermediary organic N compounds like glutamate. The NH_4_^+^ released follows the same pathway as NH_4_^+^ taken up directly (Fig. 2). The NH_4_^+^ in the cytosol is used to synthesize glutamine, which is transported into the plastid and undergoes further assimilation by the GS/GOGAT cycle, generating glutamate (Fig. 2). Glutamate generated in the cytosol by the catabolism of the transported ON can also enter the GOGAT cycle within the plasmid. In both cases, glutamate is subjected to further aminotransferase reactions to produce other AAs or cycled back to glutamine by GS_1_ (cytosolic glutamine synthetase) and GS_2_ (plastidial glutamine synthetase) (Fig. 2). Nitrate transported into the cytosol undergoes conversion to NO_2_^−^ that is transported into the plastid for further reduction to NH_4_^+^ and entry into the GOGAT cycle.

The steps involved in utilizing IN after uptake—such as NO_3_^−^ reduction, pH regulation, as well as the spatial and temporal partitioning of NO_3_^−^ reduction—increase the costs of NO_3_^−^ utilization relative to NH_4_^+^ (Penning de Vries et al. 1974; Zerihun et al. 1998). Furthermore, expulsion of H^+^ produced from NH_4_^+^ assimilation in an energy requiring process (Raven 1986). The uptake and assimilation of ON by plants requires less energy compared to IN, because N is already in a reduced form. As such, assimilating ON into other compounds such as proteins has a lower C cost than IN, due to the C atoms already contained in the ON compound. This ‘C bonus’ makes it more profitable for plants to assimilate ON than IN (Franklin et al. 2017). Earlier work estimated that the total cost of synthesizing proteins and AAs from NO_3_^−^ is at least twice as expensive as that from Gln (Penning de Vries et al. 1974; Zerihun et al. 1998). However, one must also consider the energy costs associated with ON uptake when determining if there is an overall net C benefit. Franklin et al. (2017) inferred that lower ON assimilation costs may outweigh the higher costs of ON uptake (i.e., N uptake per root biomass), therefore providing a net energy benefit relative to that of IN uptake and assimilation.

## How might organic nitrogen uptake and assimilation increase crop nitrogen use efficiency?

**Fig. 3:**
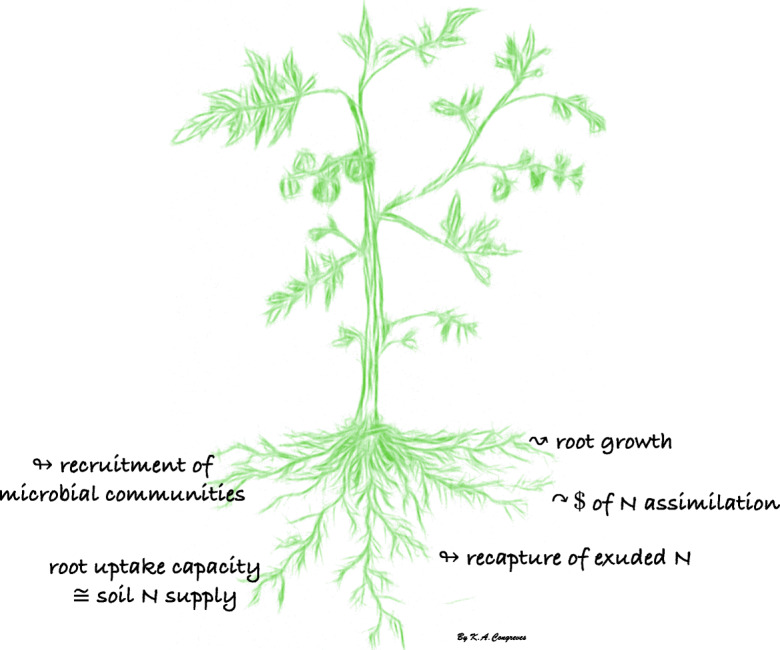
Proposed ways that soil organic N uptake and assimilation by plants may regulate nitrogen use efficiency in agricultural cropping systems: increasing root growth, lowering the cost of uptake, recruiting N-cycling microbes, recapture of exuded N, and by matching the root uptake capacity with soil N fluxes

We propose several mechanisms by which plant uptake and assimilation of soil ON may increase crop NUE: (i) lower energy costs, (ii) increasing root growth, (iii) recruiting N cycling microbial communities, (iv) re-uptake of leaked N compounds, and (v) matching the soil N supply (flux towards the roots) with root N uptake capacity (Fig. 3). For the purpose of this paper, we adopt the agronomic definition of NUE which is conceived as the amount of biomass (specifically yield) produced per unit of available N (or added N, as fertilizer)—however, there are several other ways to define NUE.

Based on the cost differences of plant ON vs. IN uptake and assimilation, it is reasonable to assume that the uptake and assimilation of ON compounds should increase plant NUE. Energy savings accrued by the provisioning of C via ON sources compared to IN forms might enable plants to support a higher growth rate and overall biomass accumulation. From a modelling study (Franklin et al. 2017), it has been inferred that plant growth rates are higher when plants assimilated ON compared to IN, and this growth rate advantage persisted as IN availability increased—even up to the point where the availability of ON was 70 % lower than IN.

When plants allocate more growth towards their roots rather than aboveground parts, it increases the capacity for N uptake by reaching a larger volume of soil, thereby potentially leading to improved NUE. In contrast to IN, much less is known about root system architecture in response to ON dynamics, but a review by Nacry et al. (2013) assembled a few studies that point towards a bushier and more branched root system when the plant relies on soil ON sources. Likewise, others have observed enhanced root growth when the plant relies on ON sources for uptake and assimilation, rather than IN sources (Cambui et al. 2011; Jiao et al. 2018; Franklin et al. 2017) estimated that—under conditions of equal growth rates—the root:shoot ratio was three times higher, and that the N productivity (i.e., the growth rate per plant N) was 20 % higher when plants utilized ON than IN. Increasing the root:shoot ratio likely favours the allocation of assimilates towards the root system, an outcome that would reduce the demand for N and offer NUE improvements. Whether or not this comes at the cost of yield should be further explored.

Plant uptake of soil N compounds is intricately related to the activity of microorganisms. Plants acquire N sources, including ON compounds, by recruiting microorganisms through mutualism and supporting multitrophic interactions. For example, plant symbioses with arbuscular mycorrhizal fungi supply not only IN but also ON, and transfer this N to the plant (Hodge et al. 2001). Also, mucilage secreted by the roots of an indigenous corn variety aid in N nutrition by way of shaping the microorganism activity in the surrounding rhizosphere, and even by recruiting N fixing bacteria (Van Deynze et al. 2018). Plants also facilitate priming of soil organic matter decomposition by shaping the activity of microorganisms in the rhizosphere. The release of root exudates can influence soil N cycling and N supply to plants—and it might even be an evolutionary strategy where the plant-derived C benefits the microbes, who in turn benefit the plant hosts through enhanced breakdown of ON pools, supplying N to plants (Moreau et al. 2019). Because plants influence the activity of microorganisms in the rhizosphere, and microorganisms regulate the forms of N in the soil (and N loss) and the N supply to plants, then crop NUE is thereby also regulated.

Simultaneous root uptake and efflux of ON compounds indicates that plants can salvage or recapture N exuded from roots (Warren 2015). One interpretation of this phenomenon is that ON uptake does not largely contribute to plant N acquisition but instead serves as a recapture mechanism for taking up N that was “leaked out” of roots cells; hence recouping the cost of N loss (Jones et al. 2005). If the secreted-ON compounds were not recaptured by the plant, they would represent a costly loss of N from the plant—providing no further direct value towards crop growth and therefore decrease crop NUE. In this view, the recapture of secreted-ON would serve to counteract the decrease in NUE, or in other words to maintain plant NUE. In weighing the two explanations for plant ON uptake—those being, a contribution towards plant N nutrition or a mechanism of retrieving leaked N—Warren (2015) concluded that the ON uptake by plants likely serves more so as a general contribution towards plant N nutrition, rather than a mere recapture mechanism. This conclusion was supported by the observation that plants took up a broad suite of ON compounds present at *very low* (sub-micromolar) concentrations, and that these concentrations similarly occur in in soil solution. Regardless of the reason for recapturing secreted-ON compounds, whether to counteract loss or to contribute to plant nutrition, the outcome serves to maintain or increase plant NUE, respectively.

In highly fertilized cropping systems, soil IN fluxes can be much higher than the plant root capacity for IN uptake (Brackin et al. 2015). Instead under such conditions, the fluxes of soil ON towards plant roots are better matched the root capacity for ON uptake (Brackin et al. 2015). The low NUE metrics that are typically observed in fertilized agricultural cropping systems could be indicative of a mismatch between soil IN fluxes and plant N uptake capacities. Better matching root N uptake capacities with the soil N fluxes may improve crop NUE. Especially in cropping systems with a legacy of frequent and excessive inorganic fertilizer applications, soil ON fluxes may be better aligned with plant ON uptake and represent a mechanism for improving crop NUE.

Crop varieties have shown sufficient differences in the uptake of ON to warrant selection of this trait in breeding programs aimed at improving NUE (Reeve et al. 2009). By better understanding the contribution of ON to crops, and by designing agricultural management strategies focused on plant uptake of soil ON sources, enhanced crop NUE might someday be realized.

## The current model of crop nitrogen nutrition

The traditional model of N nutrition focuses heavily on IN dynamics, but food crops may access a wider array of soil N sources than previously assumed. Although the quantitative role of ON remains unknown, the current model suggests that soil ON compounds are a significant and direct contributor to plant N nutrition, even in an agricultural context. Several mechanisms are proposed that increase crop NUE (Fig. 3) and should be used to direct future research. Expanding this area of research is needed to better understand plant N nutrition and to move towards improving NUE in cropping systems.
